# Sorption and desorption of Cr(VI) ions from water by biochars in different environmental conditions

**DOI:** 10.1007/s11356-014-3752-4

**Published:** 2014-11-08

**Authors:** Aleksandra Tytłak, Patryk Oleszczuk, Ryszard Dobrowolski

**Affiliations:** 1 Department of Environmental Chemistry, Faculty of Chemistry, Maria Curie-Sklodowska University, Maria Curie-Skłodowska Square 3, 20-031 Lublin, Poland; 2 Department of Analytical Chemistry and Instrumental Analysis, Faculty of Chemistry, Maria Curie-Sklodowska University, Maria Curie-Skłodowska Square 3, 20-031 Lublin, Poland

**Keywords:** Sorption, Chromium, Biochar, Environmental conditions, XPS, SEM-EDS

## Abstract

In the present research, the potential of two biochars produced by the thermal decomposition of wheat straw (BCS) and wicker (BCW) for Cr(VI) ions removing from wastewater was investigated. The pH and the presence of chlorides and nitrates were also investigated. The Freundlich and Langmuir models were applied for the characterization of adsorption isotherms. The Langmuir model has better fitting of adsorption isotherms than the Freundlich model. The sorption process can be described by the pseudo second-order equation. The optimal adsorption capacities were obtained at pH 2 and were 24.6 and 23.6 mg/g for BCS and BCW, respectively. X-ray photoelectron spectroscopy (XPS) studies confirmed that Cr(III) ions were the most abundant chromium species on the biochars’ surface. The results indicated that the sorption mechanism of Cr(VI) on biochar involves anionic and cationic adsorption combined with Cr(VI) species reduction.

## Introduction

Chromium occurs in the environment in natural forms as a ferric chromite (FeCr_2_O_4_), crocoite (PbCrO_4_), and chrome ochre (Cr_2_O_3_) (Mohan and Pittman 2006). Moreover, it is delivered into the environment from anthropogenic sources which are caused by the wide exploitation of chromium in the industry. This element and its compounds have applications in electroplating, metal finishing, petroleum refining, magnetic tapes, pigments, leather tanning, wood protection, chemical manufacturing, brass, electrical and electronic equipment, nuclear power plants, and catalysis (Kuo and Bembenek 2008; Gottipati and Mishra 2010). Chromium contaminants released by these industries are input into the soil, surface water, drinking water, and groundwater, and afterwards pass through the cell membrane of the living organism and to the food chain to the human body.

The most common species of chromium present in the environment are hexavalent chromium—Cr(VI) and trivalent chromium—Cr(III). Different states of valence affect their contrary properties such as toxicity, bioavailability, and mobility (Kotaś and Stasicka 2000). Cr(VI) is toxic, mutagenic, carcinogenic, teratogenic on biological systems, soluble, and mobile; moreover, it is thermodynamically metastable in the soil and exists in anionic and neutral species as a chromates CrO_4_^2−^ and Cr_2_O_7_^2−^ and bichromates HCrO_4_^−^, H_2_CrO_4_, and HCr_2_O_7_^2−^ (Choppala et al. 2012). Cr_2_O_7_^2−^ forms predominantly in a low pH and high chromium concentration conditions, whereas CrO_4_^2−^ are the most abundant forms at pH higher than 6.5 (Mohan and Pittman 2006). Cr(III) is nontoxic and ranked as a micronutrient because of its being an essential component of the human diet and having influence on fat, glucose (GFT, glucose tolerance factor), and cholesterol metabolism of mammals (Kimbrough et al. 1999). Cr(III) forms a stable inorganic ion as [Cr(H_2_O)_5_]^3+^ and hexacoordinate complexes (Dhal et al. 2013).

Considering the different properties of chromium species and their impact on the living organisms, especially with regards to their identification, the determination and effective reduction of Cr(VI) to Cr(III) have become recently the main subject of many studies (Kimbrough et al. 1999; Mohan and Pittman 2006; Dobrowolski and Otto 2010).

The concentration of the Cr(VI) input to the environment from industrial applications must be reduced to below 0.05 mg/L before discharging into the surface water, as is established by the US EPA (Baral and Engelken 2002). To reduce Cr(VI) concentration in water, different techniques usually are used, e.g., chemical precipitation, ion exchange, membrane separation, utrafiltration, sedimentation, and adsorption. These techniques are not free from disadvantages, e.g., high cost, low efficiency, low selectivity, high-energy requirements, and secondary toxic waste generation. Adsorption of contaminants onto activated carbon (AC) because of its high efficiency and simplicity of design is still one of the most frequently used methods (Mohan and Pittman 2006). The highly developed porosity, extended surface area, microporous structure, high adsorption capacity, and high degree of surface reactivity make the AC a good sorbent for wastewater treatment. However, there is a significant limitation to the application of these materials: commercial ACs are expensive. Because of their high prize, the necessity in the preparation of low-cost carbon materials has become an important issue for many researchers (Deveci and Kar 2013).

An interesting replacement of AC can be the biochar (BC). BC is a special type of charcoal produced by the thermal decomposition of biomass under relatively low temperature (<700 °C) and limited oxygen conditions (Oleszczuk et al. 2013). Due to the specific surface area and high sorption capacity in regard to heavy metals ions, the biochar could be used as an effective adsorbent of these contaminants (Beesley et al. 2011; Kołodyńska et al. 2012). The presence of many functional groups on the biochar surface e.g., carboxylic, alcohol, and hydroxyl, makes an opportunity to form complexes between these groups and heavy metal ions (Tang et al. 2013). Despite of the fact that BCs have become the subject of many studies recently, there is still not enough comprehensive information about their potential applications in the removal of Cr(VI) from water. Because of the growing chromium pollution, there is a necessity to establish a method which can use satisfactory biochar properties for the selective chromium removal.

The aim of this study was the evaluation of Cr(VI) adsorption onto two different biochars as a tool of reduction and immobilization of its toxic forms from aqueous media. The basic parameters having an influence on adsorption capacity were determined. The adsorption capacities were studied based on the initial runs of the adsorption isotherms. Taking into account further practical applications of biochars in water treatment, the influence of chlorides and nitrates on adsorption ability was also investigated.

## Materials and methods

### Biochar characterization

Biochars were produced by the thermal decomposition of biomass at a temperature range from 350 °C (start of combustion) to 650 °C (temperature of maximal combustion) in limited oxygen conditions (1–2 %). BCs were produced from a wicker (BCW) and a wheat straw (BCS) and provided by Fluid SA (Sędziszów, Poland) and Mostostal Sp. z. o. o. (Wrocław, Poland), respectively.

The properties of biochars were studied by the standard methods, and the results are presented in Table 1. The pH values were obtained potentiometrically in 1 mol/L potassium chloride after 24 h in the liquid/soil ratio of 10. The cation exchange capacity (CEC) and available forms of phosphorous, potassium, and magnesium were determined according to procedures for soil analysis recommended by van Reeuwijk (1992). The total organic carbon content (TOC) was measured using TOC-VCSH (Shimadzu) with solid sample module (SSM-5000). The total nitrogen (N_t_) was determined by the Kjeldahl’s procedure without the application of Dewarda’s alloy (Cu-Al-Zn alloy reducer of nitrates and nitrites).

**Table 1:** Physicochemical properties of BCS and BCW biochars

Biochar	pH	CEC	Available forms of			Elemental composition				Ash	*S*_BET_	PV
			P_2_O_5_	K_2_O	Mg	C	H	N	O			
BCS	9.9	530	540	2,824	163	54	1.8	0.9	2.3	41.0	26.3	0.026
BCW	9.1	143	122	772	32	70	3.2	0.8	17.9	8.6	11.4	0.0061

pH in KCl, *CEC* the cation exchange capacity (mmol/kg), *P*_*2*_ *O*_*5*_ *, K*_*2*_ *O, Mg* available forms of phosphorous, potassium, and magnesium (mg/100 g), *CHNO* the contribution (%) of carbon, hydrogen, nitrogen, and oxygen, *Ash* ash content (%), *H*/*C* ratio of hydrogen to carbon, *(O + N)/C* polarity index, *O*/*C* ratio of oxygen to carbon, *S*_BET_ specific surface area (m^2^/g), *PV* pore volume (cm^3^/g)

The SEM studies were performed on Tesla BS-301 microscope Quanta 3D FEG operating at 15.0 keV. The FTIR/PAS spectrum of the biochars was recorded by means of the Bio-Rad Excalibur 3000 MX spectrometer equipped with photoacoustic detector MTEC 300 with helium atmosphere in a detector at RT over 4,000–400 cm^−1^ range at resolution of 4 cm^−1^ and maximum source aperture. The obtained spectrum was normalized by computing the ratio of a sample spectrum to the MTEC carbon black standard spectrum.

For analyzing the structure of used BCs, low-temperature (77.4 K) nitrogen adsorption-desorption isotherms were obtained using Micromeritics ASAP 2405N adsorption analyzer. On the basis of the standard BET method, the specific surface areas *S*_BET_ were calculated. The CHN elemental analyzer (Carlo-Erba NA-1500) via high-temperature catalyzed combustion, followed by infrared detection of resulting CO_2_, H_2,_ and NO_2_, was used for determination of carbon, hydrogen, and nitrogen content in BCs.

### Batch sorption experiment

The standard stock solution of Cr(VI) (520 mg/L) was prepared by proper dissolution of K_2_Cr_2_O_7_ powder (POCH, Gliwice, Poland) in redistilled water. The calibration curve was established using the standard solutions of Cr(III) prepared in 2 mol/L HNO_3_ by dilution from 1,000 mg/L stock solution (Merck, Darmstadt, Germany). Moreover, Suprapur nitric acid (65 %) (POCH, Gliwice, Poland), Suprapur hydrochloric acid (36 %) (POCH, Gliwice, Poland), sodium chloride (POCH, Gliwice, Poland), and potassium nitrate (POCH, Gliwice, Poland) were used. Sodium hydroxide and hydrochloric acid solutions were used for pH adjustment.

The effect of time on the adsorption of Cr (VI) ions onto BCS and BCW vs. time intervals up to 28 h at the initial concentration of Cr(VI) at the level of 100 mg/L and the solid materials’ mass around 0.2 ± 0.03 g at pH of 2 were investigated. The adsorption capacities of Cr(VI) onto the biochars were determined by studying the initial runs of adsorption isotherms. The equilibrium isotherms were obtained at initial pH of 2, Cr(VI) concentrations from 1 to 600 mg/L range, equilibrium time of 24 h, and temperature of 25 °C. The effect of pH on Cr(VI) adsorption onto the tested biochars was carried out using series of solutions, keeping the chromium(VI) concentration at 100 mg/L with varying pH values for the particular adsorption system from 1 to 12. The effect of nitrates and chlorides on the adsorption of Cr(VI) was investigated using a series of solutions of the same chromium(VI) concentration and pH values but with increasing chloride and nitrate concentrations for the particular adsorption system. The desorption experiment in relation to HNO_3_ and HCl concentrations was conducted using BCs with Cr(VI) ions adsorbed on the surface (14 mg/g for BCS and 11 mg/g for BCW). Amount of 0.008 g of BCs was mixed with 2 mL acid solution and agitated in a shaker at room temperature for 24 h.

The measurements of the chromium concentration in the studied adsorption system were performed using flame atomic absorption spectrometer VARIAN Spectra AA-880 (Carl Zeiss, Jena, Germany). A hollow cathode lamp (Varian) was used for the measurement of chromium equilibrium concentration with a lamp current of 7 mA. The wavelength of 357.9 nm and slit width of 0.2 nm were selected for the measurement. Moreover, the values of acetylene and air flow, the basic spectrometer parameters, were 2.9 and 13.5 L/min, respectively. The limit of Cr detection was at the level of 0.14 mg/L.

The optimization of Cr(VI) ions adsorption onto BCs was conducted at 25 °C. Particular measuring points were acquired for the adsorption system as follows: 50 mL of chromium(VI) solution and 0.2 g of biochar. The equilibrium adsorption value in the solid material *a* (mg/g) was calculated from the following equation:

1 $$ a=\frac{\left({c}_{\mathrm{i}}-c\right).V}{m} $$

where *c*_*i*_ is the initial concentration of Cr(VI) (mg/L), *c* is the equilibrium concentration of Cr(VI) (mg/L), *V* is the volume of the solution (L), and *m* is the mass of the solid material (g).

### Data analysis

Pseudo first-order equation and pseudo second-order equation were used for recognizing the reaction order of obtained Cr(VI) sorption kinetics onto the examined biochars. Kinetic data were fitted to the following equations:

2 $$ \ln \left({a}_{\mathrm{eq}}-{a}_t\right)= \ln {a}_{\mathrm{eq}}-{k}_1t $$

3 $$ \frac{t}{a_t}=\frac{1}{k_2{a}_{\mathrm{eq}}^2}+\frac{1}{a_{\mathrm{et}}}t $$

where *a*_*t*_ is the amount adsorbed (mg/g) at time *t*, *a*_eq_ is the amount adsorbed (mg/g) at equilibrium time, and *k*_1_ (1/min) and *k*_2_ (g/mg min) are the rate constants of the pseudo first-order equation and pseudo second-order equation, respectively.

Two nonlinear isotherm models (Freundlich and Langmuir) were tested to fit the experimental data: the Langmuir model

4 $$ a={a}_m\frac{{\left(K{c}_{\mathrm{eq}}\right)}^n}{1+{\left(K{c}_{\mathrm{eq}}\right)}^n} $$

and Freundlich model, in which the linear form can be written as follows:

5 $$ \ln a= \ln \left({a}_{\mathrm{m}}K\right)+n \ln c $$

where *a* is the Cr(VI) ions’ adsorbed amount (mg/g) onto BCs at the equilibrium of Cr(VI) chromium concentration *c* (mg/L), *a*_m_ is the maximum adsorbed amount needed to form a monolayer on adsorbent surface (sorption capacity), *K* is the Langmuir constant, and *n* (0 ≤ *n* ≤ 1) characterizes the quasi-Gaussian energetic heterogeneity of the adsorption system.

## Results and discussion

### Biochar properties

The CEC values for BCS and BCW are distinctly different and equal to 530 and 143 mmol/kg, respectively (Table 1). For BCS, K^+^ ions dominated in the sorptive complex, next to Ca^2+^, Mg^2+^, and Na^+^. BCW ions demonstrate a different order. The Ca^2+^ ions were predominant, followed by K^+^, Na^+^, and Mg^2+^. Considering the availability of the selected forms of elements, the highest concentration was determined for potassium, which is lower for phosphorous and magnesium, without regard to the biochars’ type. BCW has higher H/C, (O + N)/C, and O/C ratios than BCS. The lowest H/C ratio for BCS was associated with the aromatic structure, with BCS having the higher carbonization level than for BCW. Moreover, comparing the O/C ratio values, it can be claimed that BCW material presents more hydrophilic structures; in addition, the highest (O + N)/C ratio, named the polarity index, indicates the higher polarity for this material. Furthermore, the BCs studied have different values of the specific surface area, 26.3 and 11.4 m^2^/g for BCS and BCW, respectively. This parameter is correlated with the pore area value, which is also significantly higher in the case of BCS.

**Fig. 1:**
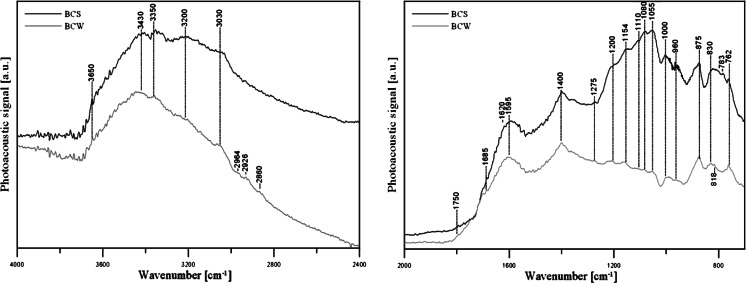
FTIR/PAS spectrum of BCs used in the study: *1* 4,000–2,400 cm^−1^ range and *2* 2,000–700 cm^−1^ range

The IR spectra of examined biochars (Fig. 1) are complicated because of the presence of multiple bands in a wide range of wavenumbers, which is also in correlation with the amount of functional groups on the surface of the biochar. At about 3,650 and 3,202 cm^−1^ in BCS, the spectra have centered band characteristics for stretching vibration of hydrogen-bonded hydroxyl groups of alcohols and carboxylic acids, respectively. In the same spectrum, the band being in correlation with hydroxyl groups of water at about 3,433 cm^−1^ can be seen. At about 3,354 and 3,358 cm^−1^ in the both spectra, the bands attributed to the primary amine groups’ presence on the biochars’ surface can be seen. The vibration at 3,068 cm^−1^ in BCW spectrum is associated with vinyl and acrylic C-H groups. The band at about 3,034 cm^−1^ is attributed to the =C-H stretching of aromatic compounds. The aliphatic C-H bands at the region 2,867–2,932 cm^−1^ can be found in the BCW spectrum, but there are no adsorption bands of those types in BCS spectrum. The bands at 1,751 and 1,685 cm^−1^ in the BCS spectrum and the similar band at 1,701 cm^−1^ in the second biochar spectra are the adsorption bands of C=O. The vibration at 1,595 cm^−1^ is associated with C=O in ketones, carboxylates, and quinones or with the C=C stretching in the aromatic components [30]. The bands in the range 1,400–1,050 cm^−1^ could be correlated with C-O and O-H presence in carboxylic acids (band at 1,356 cm^−1^) and phenols or C-O-C in ethers. The bands in the region of 1,200–1,000 cm^−1^ are attributed to the C-H deformation in the aromatic structures. The bands centered at 1,055, 957, and 783 cm^−1^ can be responsible for Si-O vibrations of inorganic materials in BCS [31]. The bands at 874 and 873 cm^−1^ in BCS and BCW spectra, respectively, can be associated with C-H bending in aromatic compounds.

### Sorption kinetics

**Fig. 2:**
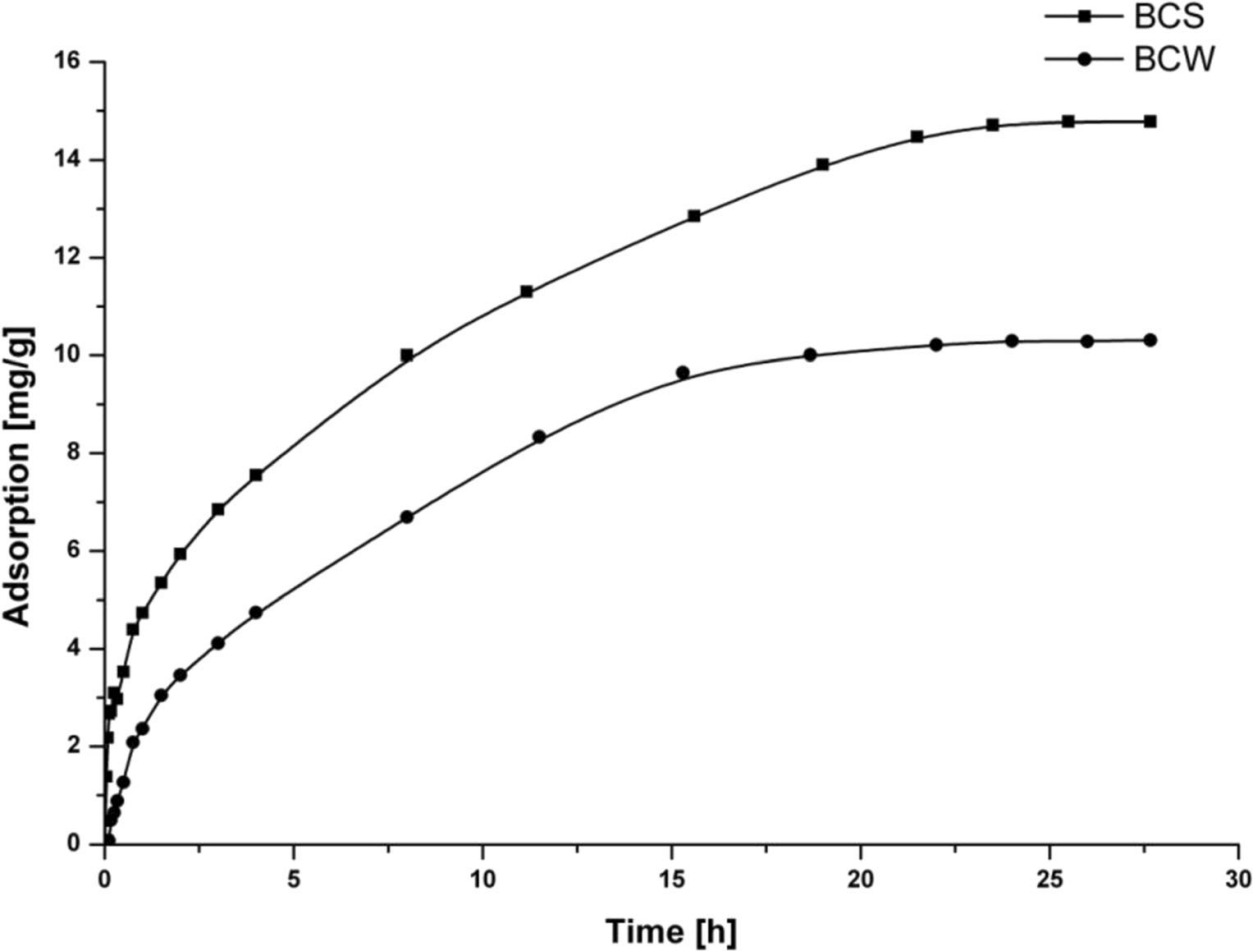
Adsorption kinetics of Cr(VI) ions onto BCS and BCW, *m* = 0.2 g, *V* = 50 mL, *C*_Cr(VI)_ = 100 mg/L, pH = 2, *T* = 25 °C

Cr(VI) demonstrated relatively fast sorption kinetics on tested biochars (Fig. 2). The equilibrium adsorption of Cr(VI) onto BCW was reached quicker than onto BCS. The equilibrium time was achieved after 21.5 h for BCS and 18 h for BCW. It is thought that the adsorption of Cr(VI) onto BCs proceeds with surface reduction of chromium(VI) to chromium(III) in the form of aqueous complex [Cr(H_2_O)_5_]^3+^ and adsorption of this complex on the biochar surface (Dobrowolski and Otto 2010). The experimental data suggest that 18 h (BCW) and 21.5 h (BCS) will be sufficient to reach the equilibrium state of Cr by tested biochars under other adsorption conditions determined as an optimum in this research. The differences between times of reaching the equilibrium for BCs may be caused by the presence of the different functional groups, acidic and basic, on the surface of the studied BCs. There are more varied functional groups on the BCS surface then BCW (Fig. 1) which are equal with the higher quantity of adsorption centers which could bond the chromium ions. Moreover, it can be the effect of different values of *S*_BET_. BCS has higher specific surface area than BCW, 26.3 and 11.4 m^2^/g, respectively, and the equilibrium state is reached longer in the case of this biochar. In the literature data for Cr(VI) ions’ adsorption onto BCs, the time of reaching the equilibrium was longer in most cases, which was more than 24 h (Zhang et al. 2013) even up to 96 h (Agrafioti et al. 2014).

**Fig. 3:**
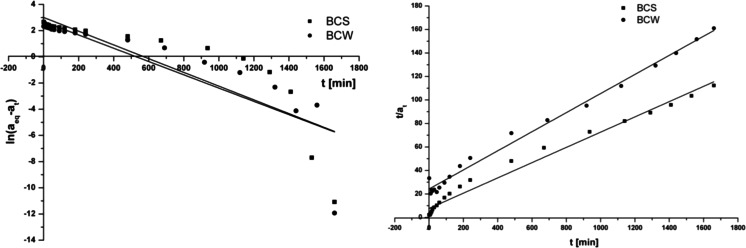
The kinetic fitting plots *1* pseudo first-order equation and *2* pseudo second-order for the BCS and BCW

Figure 3 shows the kinetic fitting plots using both equations. The linear plot of ln(*a*_eq_–*a*_*t*_) against *t* gives *k*_1_ (1/h) and *a*_eq_ values for the pseudo first-order model, whereas the plot of *t*/*a*_*t*_ against *t* gives *k*_2_ and *a*_eq_ for pseudo second-order equation. The kinetic parameters for the studied adsorption systems were calculated and shown in Table 2. The results denote that the pseudo second-order equation is better correlated with the studied adsorption system than the pseudo first-order equation. The correlation coefficients for BCS were 0.749 and 0.983 and for BCW 0.778 and 0.992 for pseudo first-order equation and pseudo second-order equation, respectively. It can be explained by chemical sorption or chemisorption processes which involve valency forces through sharing or exchanging of electrons between the adsorbate and solid material. In comparison to the previous studies, the obtained data are in agreement with most studies concerning the Cr(VI) ions adsorption (Aliabadi et al. 2012; Zhang et al. 2013). The *a*_eq_ values for the studied BCs were 14.36 mg/g (BCS) and 10.28 mg/g (BCW). Moreover, the sorption capacities calculated from pseudo second-order equation were consistent with the experimental value and were 14.78 mg/g (BCS) and 10.31 mg/g (BCW).

**Table 2:** Parameters of pseudo first-order and pseudo second-order kinetic models for Cr(VI) adsorption onto BCS and BCW biochars

Biochar	Kinetic model					
	Pseudo first order			Pseudo second order		
	*k*_1_	*R* ^2^	*a*_eq_	*k*_2_	*R* ^2^	*a*_eq_
BCS	0.0052	0.749	–	0.00059	0.983	14.78
BCW	0.0051	0.778	–	0.00039	0.992	10.31

*a*_*t*_ the amount adsorbed (mg/g) at time *t*, *a*_eq_ the amount adsorbed (mg/g) at equilibrium time, *k*_1_ (1/min) and *k*_2_ (g/mg min) the rate constants of the pseudo first-order equation and pseudo second-order equation, respectively, *R* regression coefficient

### Sorption isotherms

**Fig. 4:**
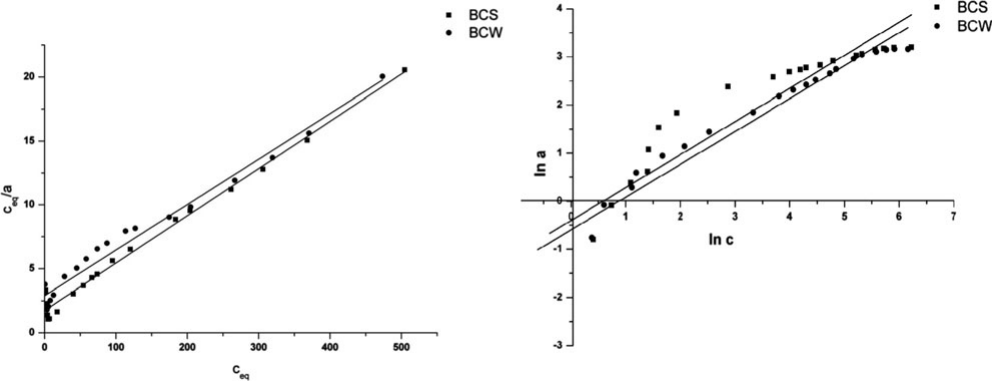
Langmuir (*1*) and Freundlich (*2*) isotherms for chromium(VI) adsorption onto BCS and BCW

The maximum sorption capacities obtained for both biochars were similar and equal to 24.6 mg/g for BCS and 23.6 mg/g for BCW. The linear dependencies obtained are plotted for the each isotherm model and are shown in the Fig. 4. In Table 3, the corresponding constants and correlation coefficients are reported. For both examined BCs, Langmuir model gave a slightly better fit and provided the best correlation to the isotherms data. The correlation coefficients were 0.990 and 0.978 for BCS and BCW, respectively. However, the studied Cr(VI) adsorption isotherms are in good agreement with the linear form of the Freundlich equation for higher concentration of chromium(VI) (Fig. 4). The decreasing of *n* value to zero causes the increasing adsorption heterogeneity. According to the data presented in Table 3, the *n* value obtained for BCS material is smaller than for BCW material, which is associated with greater energetic heterogeneity of BCS and the smaller value for BCW biochar.

**Table 3:** Langmuir and Freundlich constants and correlation coefficients

Biochar	Langmuir			Freundlich		
	*K*_L_	*a*_m_	*R* ^2^	*K*	1/*n*	*R* ^2^
BCS	0.017	28.1	0.990	0.06	1.45	0.888
BCW	0.012	28.1	0.978	0.08	1.47	0.952

*a*_m_ the maximum adsorbed amount (sorption capacity) (mg/g), *K*_L_ the Langmuir constant—the quasi-Gaussian energetic heterogeneity of the adsorption system, *R* regression coefficient, *K*, *n* empirical constants indicative of sorption capacity and sorption intensity

The greater adsorption capacity for BCS was partially correlated with higher CEC value. This effect can be also explained by the pore volume of investigated biochars. Cr(VI) ions are adsorbed on the BCs’ surface in form of [Cr(H_2_O)_5_]^3+^. The pore volume has significant influence on adsorption process of these cations because of their size. On the basis of Table 1, BCS has higher PV, which can be one of the few explanations of the higher adsorption value of this material. The transport of chromium ions through BCW pores was probably more difficult.

The O/C index should be also considered as a possible reason of higher adsorption capacity of BCS. BCS is more hydrophobic than BCW, which can make this property responsible for stronger electrostatic interaction between [Cr(H_2_O)_5_]^3+^ ions and the BCS surface.

### Effect of pH

**Fig. 5:**
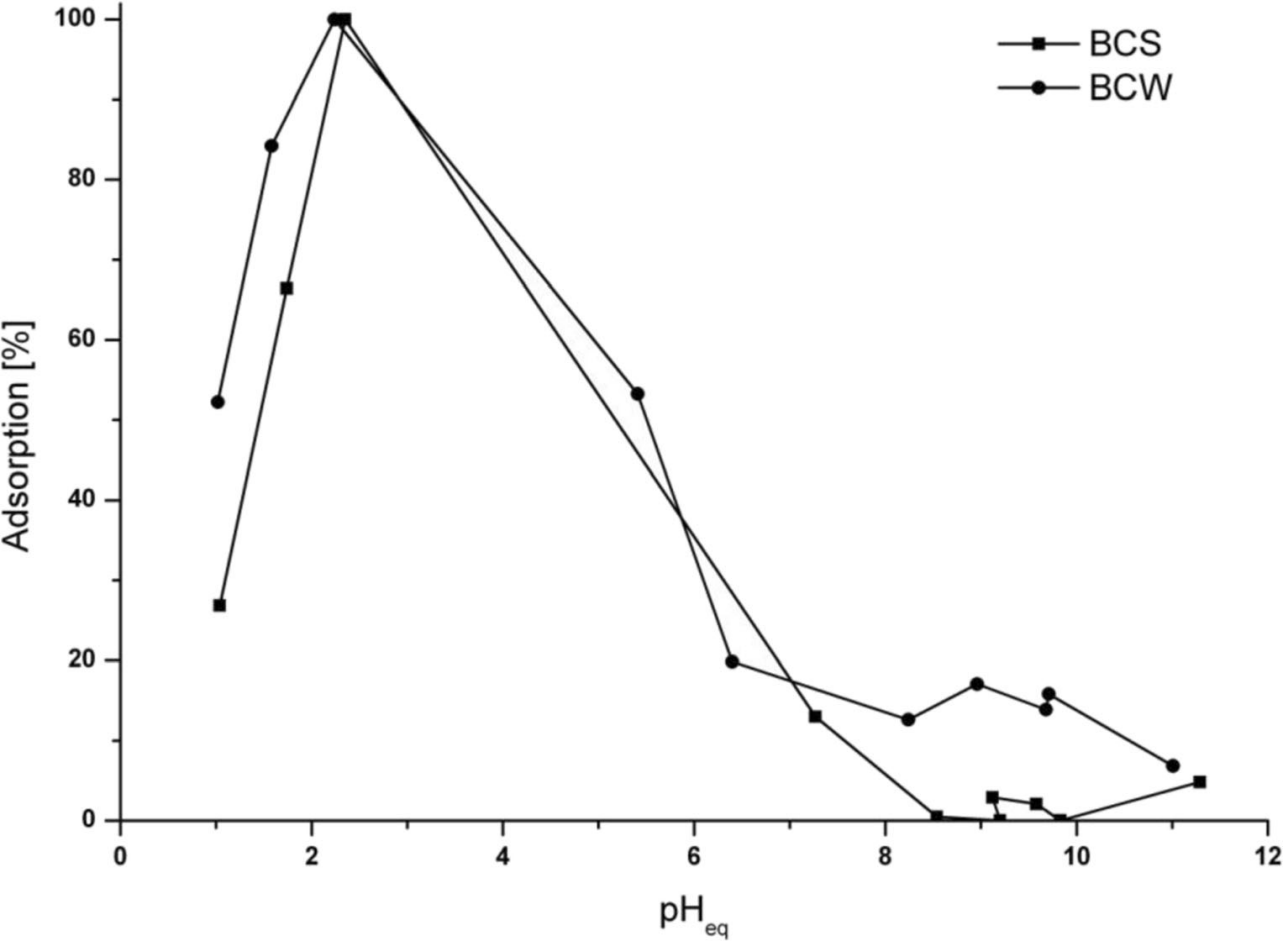
The pH influence on Cr(VI) ions’ adsorption onto BCS and BCW; *m* = 0.2 g, *V* = 50 mL, *C*_Cr(VI)_ = 5.2 mg/L, *t* = 24 h, *T* = 25 °C

It has been proved that pH is one of the most important parameters having an influence on the adsorption capacity of adsorbent for heavy metal ions removal from the aqueous solutions (Kołodyńska et al. 2012; Aliabadi et al. 2012; Deveci and Kar 2013). In Fig. 5, the adsorption abilities of chromium in an equilibrium pH are shown depending on the biochar tested. The variations in chromium(VI) sorption ability in the examined pH range may be partly related to pH dependency of the chromium species present in the aqueous solution and onto the BCs’ surface. At pH 2, Cr(III) ions exist in aqueous solution as [Cr(H_2_O)_5_]^3+^ which has associated water molecules, whereas Cr(VI) is unstable. These molecules can be exchanged with the hydroxyl ions which depend on the pH values. Changes in acidic groups present on the surface of the biochar cause the variations of pH. The maximum adsorption of Cr(VI) ions onto BCs was achieved at pH 2. For both of BCs in the pH range from 1 to 7, the same trend was observed, and the differences between biochars were only noticeable above pH 7. It is caused by the uncompleted reduction of Cr(VI) and mixed Cr(III) and Cr(VI) adsorption processes. At pH lower than 2, in acidic conditions, the reduction of Cr(VI) to Cr(III), followed by the adsorption by nonspecific sorption or surface chelation, is the main mechanism of Cr(VI) adsorption. At pH 2.0–3.2, Arslan and Pehlivan (2007) noticed that carboxylic acid centers could be appreciably deprotonated and exist in the form of COO^−^ which can bind to Cr(III) ions. Furthermore, Gardea-Torresdey et al. (2000) reported that Cr(III) can be bound to the carboxyl groups containing oxygen. In higher pH values, acidic surface groups enhanced the adsorption of Cr(III) onto the biochar. These kinds of groups were identified by FTIR (Fig. 1) and X-ray photoelectron spectroscopy (XPS) (Fig. 8) (which will be discussed later) techniques onto the BCs’ surface.

### Effect of NO_3_^−^ and Cl^−^

**Fig. 6:**
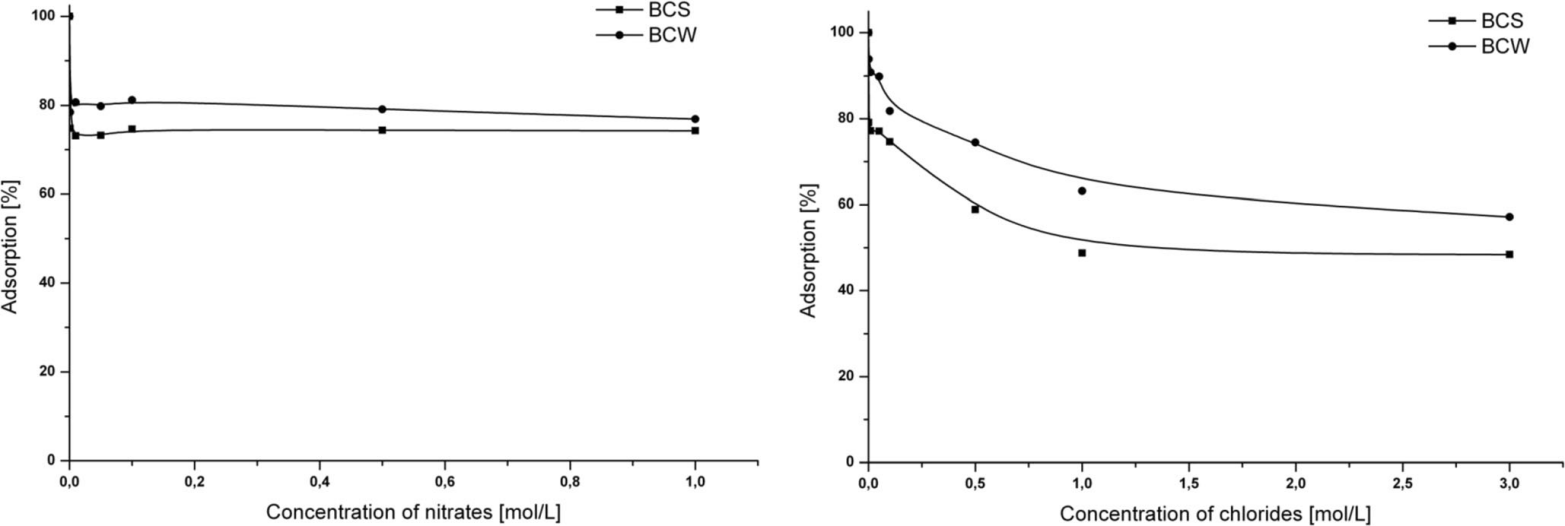
The influence of *1* nitrates and *2* chlorides on Cr(VI) adsorption onto BCS and BCW; *m* = 0.2 g, *V* = 50 mL, *C*_Cr(VI)_ = 100 mg/L, pH = 2, *t* = 24 h, *T* = 25 °C

The influence of oxidants and reducing agents on Cr(VI) adsorption ability on the biochar is important. Nitrates have quite different impacts on Cr(VI) adsorption onto BCs than chlorides (Fig. 6). In the case of nitrates, the decrease of Cr(VI) adsorption to 74 % (BCS) and 77 % (BCW) was observed at the concentration of these ions below the level of 0.001 mol/L. The concentration above 0.001 mol/L of KNO_3_ does not cause the decreasing adsorption, which acquires the constant value for both BCs. For the highest ions’ concentration, chlorides cause the decrease of the adsorption value to 49 and 57 % for BCS and BCW, respectively. These results are compared with the adsorption obtained for the Cr(VI) solution without interferences’ presence and with optimal pH value. These drastic effects can be caused by the competitive interactions between Cr(VI) and nitrates or chlorides towards biochar adsorption centers. The more drastic impact of chlorides on Cr(VI) ions’ adsorption can be explained by the fact that for examined BCs, the adsorption was nonspecific or through the diffuse layer. A competitive interaction between CrO_4_^2−^ and Cl^−^ takes place. The differences between the interferences impact on adsorption can be related to higher tendency chlorides for binding to the aliphatic group presented in the structure of BC.

### Desorption study

**Fig. 7:**
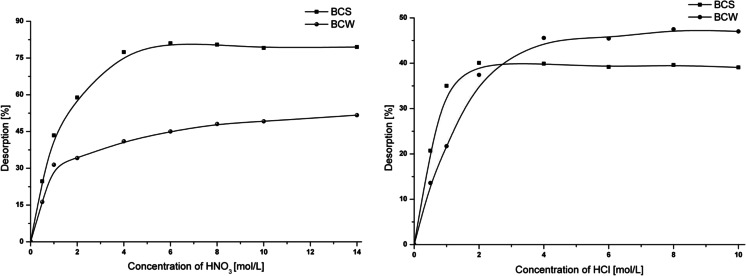
Desorption of Cr(VI) from BCS and BCW in respect to *1* nitric acid and *2* hydrochloric acid concentration; *m* = 0.008 g, *V* = 2 mL, *A*_Cr(BCS)_ = 14 mg/g, *A*_Cr(BCW)_ = 11 mg/g, *t* = 24 h, *T* = 25 °C

Batch desorption experiments were conducted in order to investigate whether chromium adsorption on investigated biochars is reversible or not. The desorption studies of Cr(VI) ions in relation to HCl and HNO_3_ concentrations were performed. The desorption kinetics of chromium forms was similar for both materials and does not exceed 20 min (Fig. 7). The maximum desorption of Cr(VI) ions from the biochars’ surface cannot be achieved even if concentrated HCl or HNO_3_ was applied. Additionally, for nitric acid as a desorptive agent, the lowest desorption is observed for BCW (51 %) and the greatest for BCS (79 %). A different effect is observed for hydrochloric acid, wherein the lowest desorption is for BCS (39 %) and greatest for BCW (47 %). This study confirmed that even the application of concentrated hydrochloric or nitric acid does not cause the total desorption of Cr(VI) ions from the biochar surface. Moreover, it should be noticed that chromium(VI) adsorption process onto BCs is irreversible, which can be caused by the surface precipitation of Cr(OH)_3_. The differences between desorption obtained in the case of both desorptive agents can be related to the fact that the adsorption of Cr(III) species proceeds by different functional groups, acidic and basic, on the BCS and BCW surfaces. Furthermore, the different structures of studied biochars (different *S*_BET_) (Table 1) can be responsible for uncompleted desorption.

### XPS study of chromium-loaded BCs

**Fig. 8:**
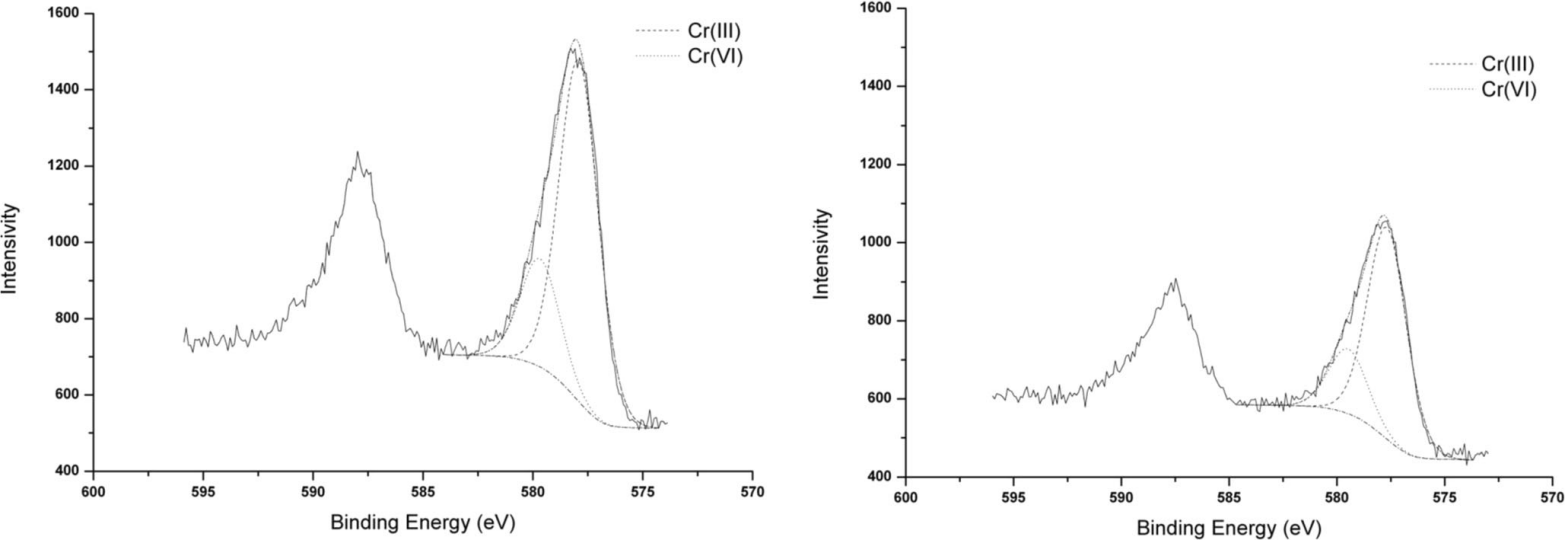
Cr2*p* XPS spectra of *1* BCS and *2* BCW

The information about the distribution of element species loaded onto the biochars’ surface and the confirmation of proposed Cr(VI) ions adsorption mechanism were obtained using XPS technique. In Fig. 8, the XPS spectrum of the Cr2*p* region is shown. In both element species, Cr(III) and Cr(VI) were detected on the BCs’ surface. The fitted peaks for the Cr2*p* region and their possible assignments implicate that the main species as a result of the chromium adsorption onto BCs are Cr(III) and Cr(VI), which represent about 76 and 24 %, respectively. It can be caused by uncompleted reduction of Cr(VI) to Cr(III) on the biochars’ surface. Significant bands at the binding energies of 577–580 were used for the identification of the chromium state of valence (Cr2*p*_3/2_ region). The bands at binding energy of 577.9 and 577.7 for BCS and BCW, respectively, indicate the presence of Cr(III) species (CrCl_3_ and Cr(OH)_3_). Furthermore, in the same Cr2*p*_3/2_ region, the bands of 579.7 and 579.5 confirmed the Cr(VI) species’ loading on the BCs’ surface (CrO_3_), in respect to BCS and BCW.

On the basis of the XPS data, it is confirmed that Cr(III) is the most abundant species on the BCs’ surface, and the main mechanism of chromium(VI) adsorption onto these materials is the surface reduction of Cr(VI) to Cr(III). This effect determines the selectivity of the applied method. After the uncompleted reduction, both chromium species are adsorbed on the BCs’ surface by oxygen-containing functional groups. On the basis of Saha and Orvig (2010) classification, the reaction of Cr(VI) ions with biomaterials can proceed by four mechanisms: (1) anionic adsorption, where Cr(VI) ions are adsorbed through electrostatic interaction, without Cr(VI) reduction by porous material; (2) adsorption coupled with reduction, where Cr(VI) is adsorbed and completely reduced by porous material to Cr(III) form; (3) anionic and cationic adsorption, where Cr(VI) is reduced by porous material to Cr(III) which is finally adsorbed onto BCs; and (4) reduction and anionic adsorption, where Cr(VI) is adsorbed and the remainder is reduced to Cr(III). Accordingly, the Cr(VI) adsorption onto BCS and BCW can proceed by mixed 1 and 3 mechanisms, because both Cr(VI) and Cr(III) species are present on the biochars’ surface.

### SEM-EDX study

**Fig. 9:**
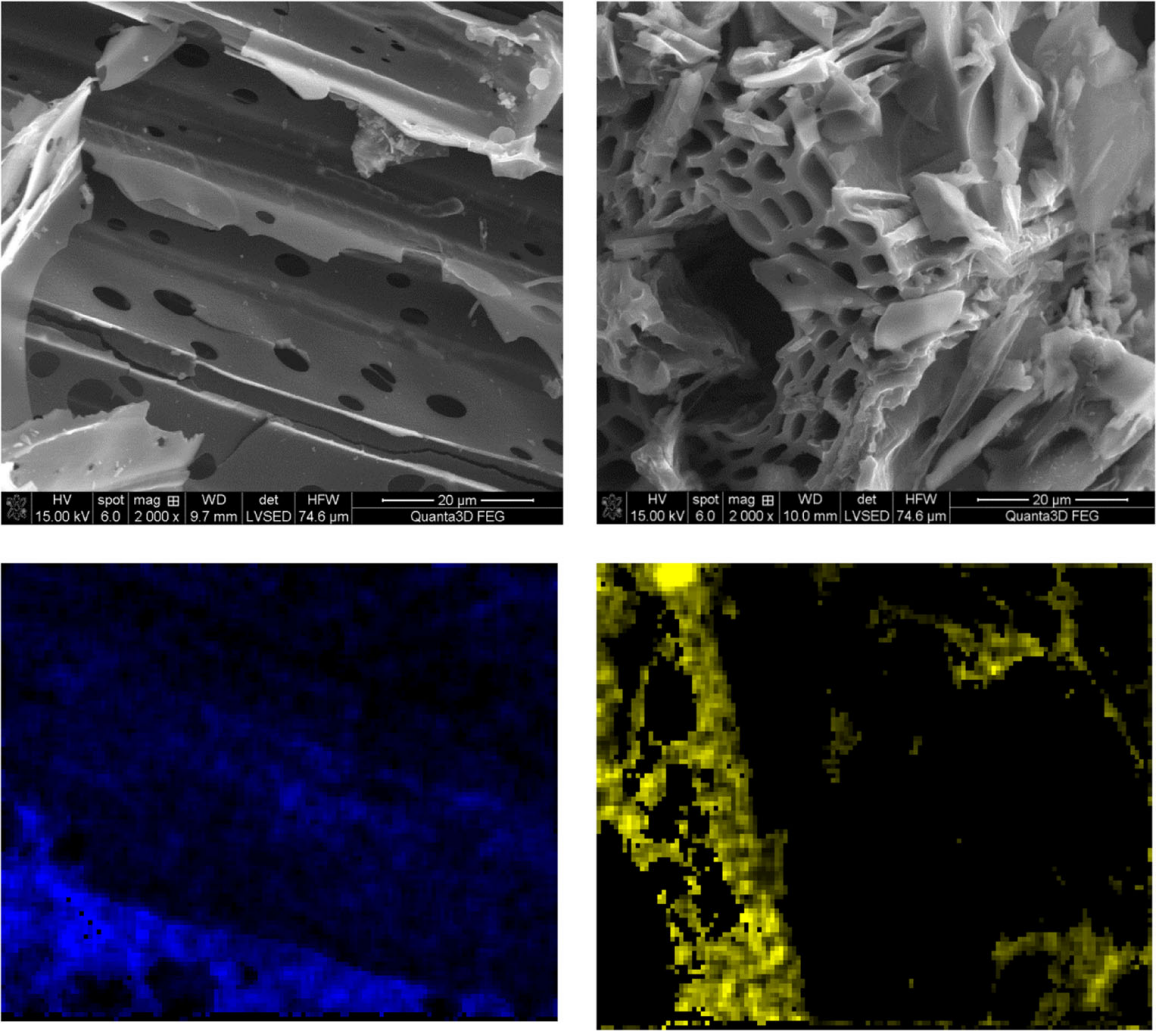
SEM of *1* BCS and *2* BCW materials used in Cr(VI) adsorption processes and X-ray elemental mapping. *Blue* and *yellow* colors show Cr loaded on particular BCs

To evaluate the surface physical morphology and the porous structure of examined biochars, SEM analysis was performed. Figure 9 presents the SEM images of chromium-loaded BCs and confirmed their porous structure. Furthermore, the Cr elemental mapping shows the presence of chromium onto biochars in the form of an irregular island on material edge. That irregular contribution of chromium loaded onto biochar surface confirms their heterogeneous structure.

## Conclusions

The tested biochars were suitable for the removal of Cr(VI) ions from wastewater. The studied BCs were characterized by similar chromium sorption capacities. The Cr(VI) adsorption process was highly pH-dependent and the pH value of 2 was optimal for Cr(VI) removal from the solution. These acidic conditions are favorable for geological sample analysis because precipitation of transition metal ions can be excluded. The obtained experimental adsorption results were successfully fitted to the Langmuir isotherm model. The partial irreversibility process and X-ray photoelectron spectroscopy data confirmed that the main Cr(VI) adsorption mechanism onto BCs is the surface reduction of Cr(VI) to Cr(III). It is worth to pointing out that the tested biochars could be used as an effective, low-cost adsorbent for Cr(VI) removal from aqueous solutions under the determined optimal experimental conditions. The disadvantage of the long-range application of these materials is the inability of repeated usage caused by uncompleted desorption of Cr species from biochars’ surface. The physicochemical improvement processes could be applied for enhancing the adsorptive properties of the studied BCs.
